# Deprivation of visual input alters specific subset of inhibitory neurons and affect thalamic afferent terminals in V1 of *rd1* mouse

**DOI:** 10.3389/fncel.2024.1422613

**Published:** 2024-10-09

**Authors:** Kashish Parnami, Anushka Surana, Vineet Choudhary, Anwesha Bhattacharyya

**Affiliations:** ^1^Amity Institute of Neuropsychology and Neurosciences, Amity University Noida, Noida, India; ^2^Department of Biotechnology, All India Institute of Medical Sciences (AIIMS), New Delhi, India

**Keywords:** retinal degeneration, primary visual cortex, GABAergic neurons, thalamocortical afferents, excitation-inhibition balance

## Abstract

Retinitis Pigmentosa (RP) is a heterogenous group of inherited disorder, and its progression not only affects the retina but also the primary visual cortex. This manifests imbalances in the excitatory and inhibitory neurotransmission. Here, we investigated if changes in cortical functioning is linked to alterations in GABAergic population of neurons and its two important subsets, somatostatin (SST) and parvalbumin (PV) neuron in *rd1* model of retinal degeneration (RD). We demonstrate marked decrease in the proportion of SST neurons in different layers of cortex whereas PV neurons were less affected. Moreover, we found reduced expression of glutamatergic thalamic afferents (VGLUT2) due to lack of visual activity. These results suggest PV neurons are likely recruited by the cortical circuitry to increase the inhibitory drive and compensate the disrupted inhibition-excitation balance. However, reduced SST expression perhaps results in weakening of stimulus selectivity. Delineating their functional role during RD will provide insights for acquisition of high-resolution vision thereby improving current state of vision restoration.

## Introduction

Humans rely primarily on their eyesight to provide information about the outside world. The neural retina is a light sensitive laminar tissue containing neurons equipped with the machinery to process light information received by the eye (Mahabadi and Al Khalili, 2024; Ptito et al., 2021). In major retinal degenerative diseases, such as retinitis pigmentosa (RP) and age- related macular degeneration, the retina undergoes deterioration leading to irreversible death of photoreceptors leaving the remaining tissue light insensitive (Zhang et al., 2021; Bhattacharyya, 2022; Jones et al., 1995). Inherited retinal degeneration, like RP is the most common cause of blindness affecting approximately 1 in 5,000 individuals in which genetic mutation lead to progressive death of photoreceptors and loss of vision (Hartong et al., 2006; Duncan et al., 2018; O'Neal and Luther, 2024). Mutation in the photoreceptor cGMP phosphodiesterase6b (*Pde6b*) gene typically manifests in loss of rod photoreceptor cells followed by the death of cones causing progressive loss of night vision and peripheral visual field (Hartong et al., 2006; Chun-Hong Xia et al., 2023; Zhang et al., 2024). PDE6B present in the outer segment of rods is an essential component of the phototransduction cascade that hydrolyzes cGMP to GMP phosphodiesterase leading to closure of cGMP gated channels and hyperpolarization of cell (Lagman et al., 2016).

Mutant mouse models of retinal degeneration (RD) are valuable tools in developing effective treatment for RP as these strains identify human pathology with same mutant genes and phenotypes (Chun-Hong Xia et al., 2023; Han et al., 2013). The *rd1* (rodless) mouse was first discovered by Keeler in 1924 having an autosomal recessive mutation in the PDE6B gene resulting in increased cGMP and calcium levels leading to rapid loss of rods beginning at P14 and complete loss by 4 weeks (Han et al., 2013; Bowes et al., 1990). The degeneration of rods subsequently creates an unstable environment contributing to the death of single layer of photoreceptors, the cones (Brunet et al., 2022). The *rd1* mouse model has given opportunities to better understand and develop innovative experimental therapies to rescue visual function for treating late-stage RP (Bhattacharyya, 2022; Lindner et al., 2022). In the past decade researchers have developed a diverse optogenetic toolkit, however, most of them had limitations in achieving high resolution vision which relies on the integrity of downstream visual circuit (Chen et al., 2020; Chen et al., 2016; Parnami and Bhattacharyya, 2023; Berry et al., 2017; Katada et al., 2023; Gauvain et al., 2021; Chen et al., 2022; Sahel et al., 2021; McGregor et al., 2022).

Reports show that progressive RD leads to alterations in the glutamatergic thalamocortical terminals (VGLUT2) in middle layers of cortex, spine distribution in lower layer (Martinez-Galan et al., 2022) and glial gene expression having implications in neural plasticity and brain function (Cornett et al., 2011). Electrophysiological studies in various rodent RD models have reported changes in the neural properties of primary visual cortex (V1), the first cortical area processing visual information (Chen et al., 2016; Wang et al., 2016; Gias et al., 2011). This includes decrease in receptive field size, diminished ability to discriminate pattern stimulus with different contrast levels and reduced orientation selectivity (Chen et al., 2016). Changes in the visual cortex is further accompanied by abnormalities in synaptic transmission in the thalamus affecting the level of excitation and inhibition (Pietra et al., 2021) with increased spontaneous activity in V1 (Wang et al., 2016).

Although previous study has shown that postnatal development of the visual cortex is not affected by RD, the negative retinal remodeling may have implications on the key players of cortical network dynamics (Himmelhan et al., 2018). Information processing in the V1 relies primarily on interactions of pyramidal neurons and diverse inhibitory neurons and it has been shown that there is a net shift of inhibition in the local circuits. Inhibitory neurons (INs) constitute 20–30% of cortical neurons and are crucial for regulating excitatory activity and network dynamics in the cortex (Song et al., 2021).

In this present study we investigated if the compensatory increase in inhibitory drive to balance the inhibition-excitation in the V1 during RD also parallel changes in two important classes of interneurons, parvalbumin (PV) and somatostatin (SST) by using immunohistochemistry and confocal microscopy. PV and SST neurons comprise ~40 and 30% of all GABAergic interneurons that target the somatic compartment and distal dendrites of pyramidal cells (Druga et al., 2023; Rudy et al., 2011). In addition, we studied if progressive deprivation of visual input affects glutamatergic signaling in the V1 of *rd1* mice. Our results show no significant changes in the overall population of GABA+ neurons although there were layer specific differences in the expression of a distinct class of inhibitory neuron. Gradual decline of sensory input results in substantial loss of SST+ neurons whereas the overall proportion of PV remain unaffected in any layers of V1. Moreover, a distinct band of VGLUT2 expression in the middle layers of V1 was observed in control mice that was absent in *rd1* mice consistent with decreased protein level. The present study provides new insights about the plausible reason for physiological alterations and facilitate improvement of impaired vision during inherited RD.

## Results

The aim of this study was to investigate whether progressive degeneration of retinal photoreceptors that causes functional decline of V1 is linked to changes in the overall population of GABAergic neurons and its two major subtypes. We investigated changes in the population of GABAergic neurons to total neurons and quantified the expression of parvalbumin (PV) and somatostatin (SST) expressing inhibitory neurons in different layers of the V1. Next, we analyzed the expression of vesicular glutamate transporter 2 (VGLUT2), by immunohistochemistry and assessed its protein level in V1 at early stage of degeneration.

### Degeneration of photoceptors does not alter laminar pattern and neuronal distribution in V1

**Figure 1:**
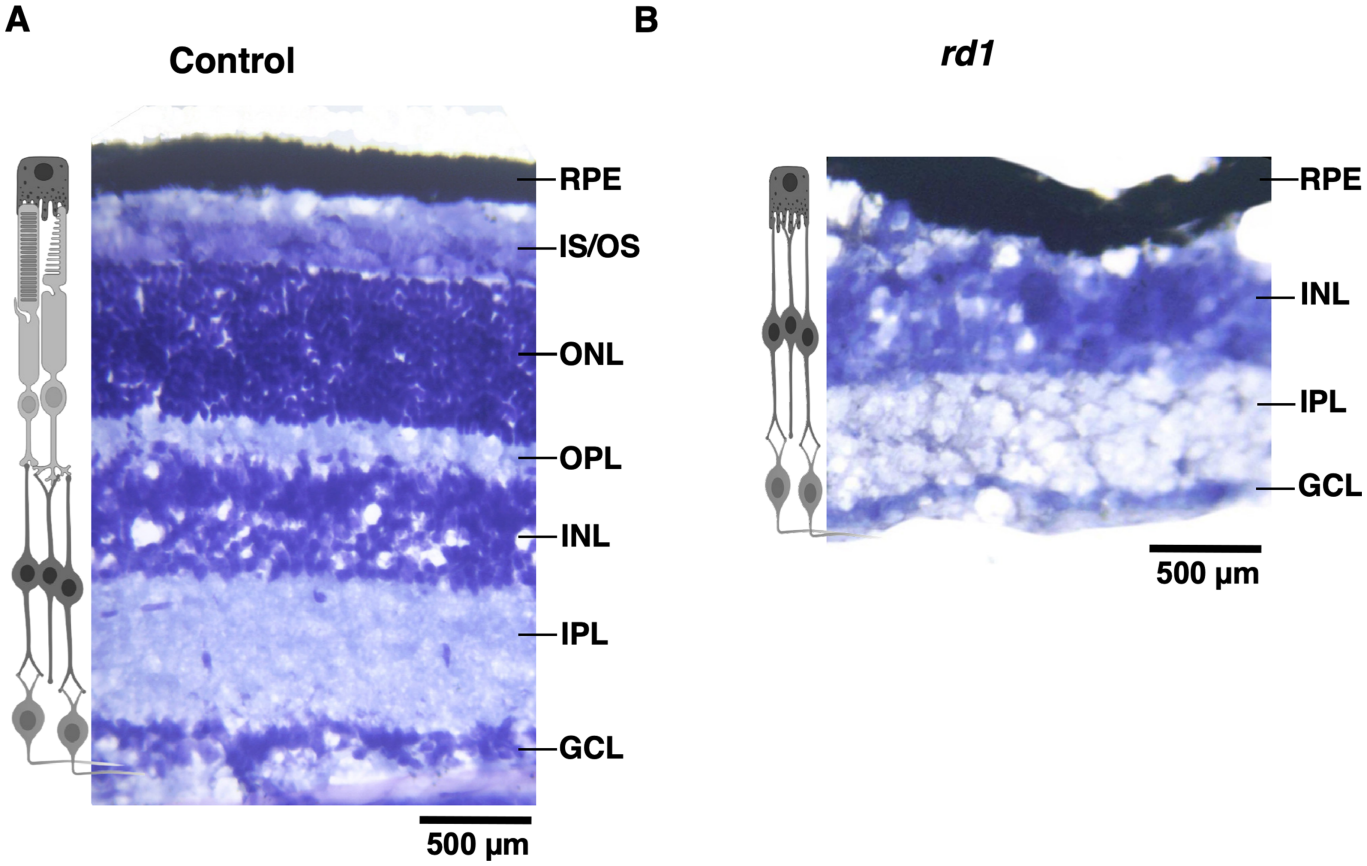
Toluidine blue staining of retinal section from C57BL/6 (control) and C3H/HeJ (*rd1*) mice at P40. The retina section of control mice has an intact laminar structure comprising of outer segment OS; inner segment (IS); outer plexiform layer (OPL); outer nuclear layer (ONL); inner plexiform layer (IPL) and ganglion cell layer (GCL) **(A)**. Progressive degeneration of the photoreceptors manifest in marked thinning of the retinal layers due to the absence of IS, ONL, and OPL **(B)**. The inner retinal layers does not exhibit any changes (Scale bar: 500 μm).

**Figure 2:**
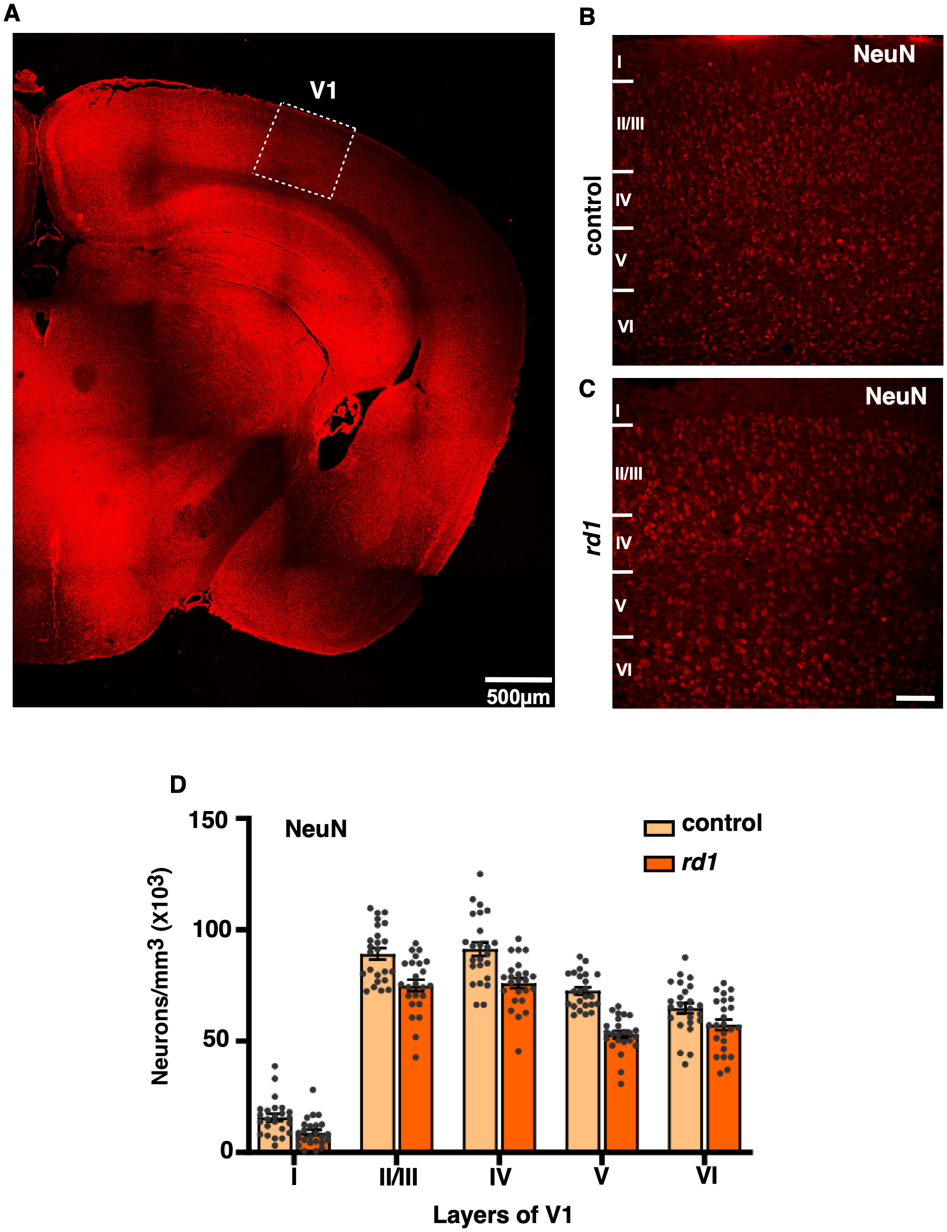
Representative coronal section of primary visual cortex (V1) immunostained with NeuN (total neuronal population) of control and *rd1* mice **(A)**. Dotted boxes highlight the primary visual cortex (V1). **(B,C)** are magnified view of V1 showing all the layers in control (B) and *rd1* **(C)** mice. The overall organization, lamination pattern and distribution of neurons are similar in both strains (*n =* 3) and is not affected by retinal degeneration (RD) **(D)** [Scale bars: 500 μm **(A)**; 75 μm **(B,C)**].

First, we visualized the morphology of retina of both C57BL6 (control) and degenerated C3H/HeJ (*rd1*) mice by performing toluidine blue staining of retinal sections. In the control retina, all the layers can be clearly observed showing normal thickness of the outer and the inner retina (Figure 1A). However, we found that *rd1* mice at an early age (P40) displayed marked thinning of the photoreceptor layer, having complete loss of the outer nuclear layer (ONL) and outer plexiform layer (OPL) compared to control (Figure 1B), consistent with previous findings (Han et al., 2013; Contreras et al., 2021). Next, we investigated if degeneration of photoreceptors affects the cortical organization in the primary visual cortex (V1). Hence, we examined coronal sections of V1 (dotted box) by visualizing laminar distribution of total neuronal population using NeuN labeling (Figure 2A). Interestingly, we found overall structural organization, laminar pattern, and distribution of neurons in V1 of *rd1* mice that resembled to control (Figures 2B,C). Quantitative analysis revealed comparable neuronal density in *rd1* compared to control in all layers of V1 and showed no significant differences (Figure 2D). These results indicate that loss of photoreceptors does not alter gross neuronal distribution across different layers of V1.

### VGLUT2 expression in the V1 is affected by RD

**Figure 3:**
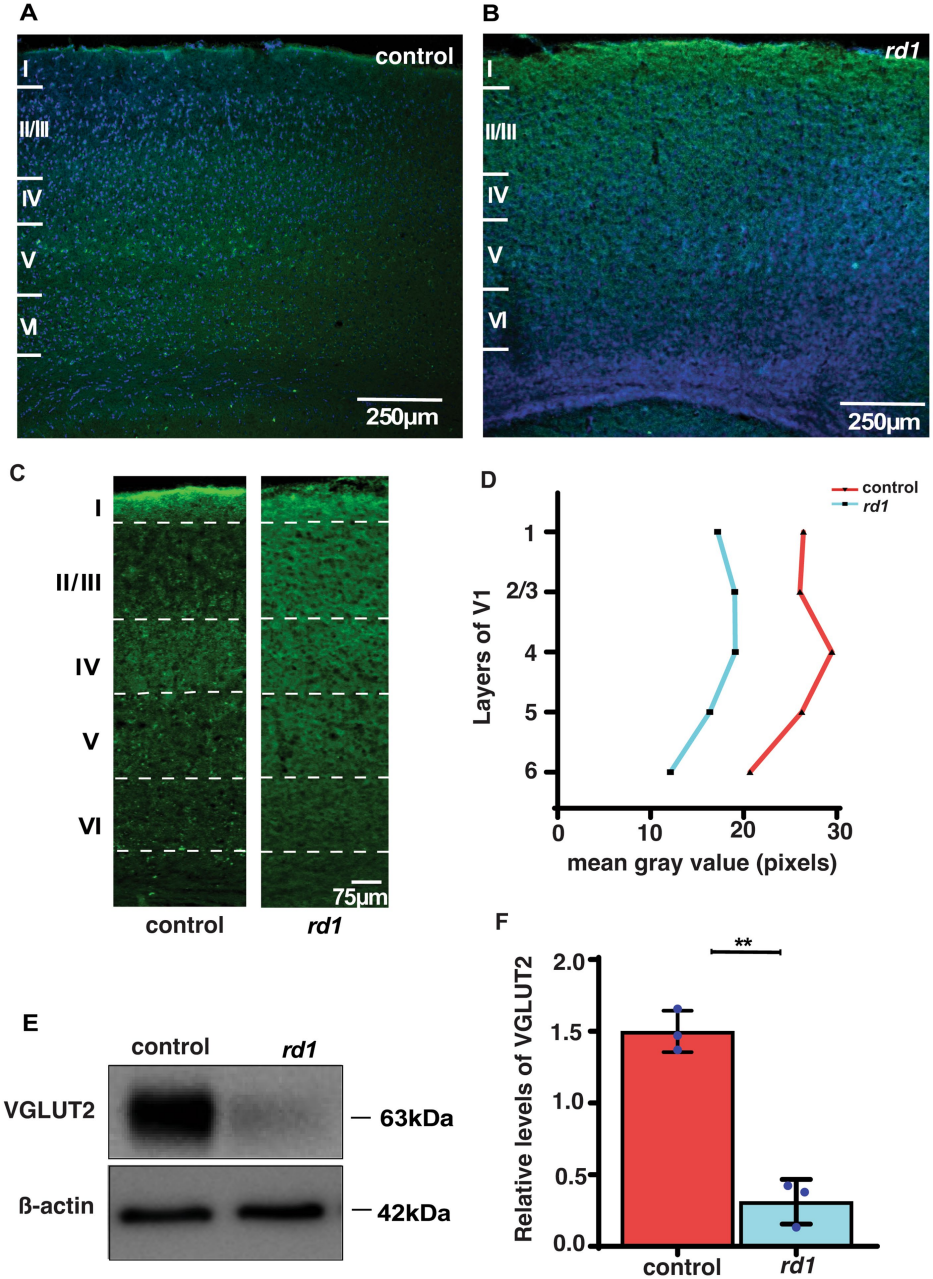
VGLUT2 immunolabeling in the visual cortex of control **(A)** and *rd1* **(B)** mice and nuclei counterstained with DAPI (blue). Magnified confocal images show a distinct band of VGLUT2 labeling in layer IV of control **(A)** but diffused in *rd1* mice **(B)**. **(C,D)** Quantification of VGLUT2 immunolabeling in control and rd1 mice in all layers of V1 by performing mean gray value determination (*n =* 3; *N =* 6 sections). Further, protein expression levels of VGLUT2 were assessed through blotting in **(E)**, with *β*-actin used as the internal standard. The graph **(F)** illustrates the percentage of VGLUT2 protein expression in both genotypes, showing significantly higher levels in control mice in comparison with *rd1* mice (*n =* 3, *t*-test, *p* < 0.01). The blue dots represent average for individual animals. The data is presented as mean ± SEM percentage [Scale bar: 250 μm **(A,C)**; 75 μm **(B,D)**]. Magnification: 10X **(A,C)** 20X **(B,D)**.

We further investigated whether early degeneration in *rd1* mice affects the expression of glutamatergic thalamic afferents from the dLGN to the middle layers in the V1. A qualitative and quantitative comparison of VGLUT2 was done in V1 of both strains. VGLUT2 is one of the isoforms of vesicular glutamate transporter that is essential for glutamate uptake and release in central synapses (Graziano et al., 2008; Juge et al., 2006). We performed single immunostaining of VGLUT2 that labels the thalamocortical afferents and counterstained with DAPI for identification of the cortical layers. At low magnification (20x), we observed enriched immunolabeled band of VGLUT2 in layer IV of control mice and modest labeling in layers V and VI (Figure 3A). In contrast, we found reduced immunolabeling of VGLUT2 in degenerated mice at P40 (Figure 3B). Next, we quantified the immunolabeling of VGLUT2 by determining mean gray value as previously described (Schindelin et al., 2012). Remarkably, we found reduced immunolabeling of VGLUT2 in all layers of V1 compared to control (Figures 3C,D). In addition, we also measured the levels of VGLUT2 by Western Blot analysis. Interestingly, we found that the expression level of VGLUT2 was markedly reduced in *rd1* mice with respect to control (*p* < 0.001) (Figures 3E,F). Taken together, we show for the first time that the expression of VGLUT2 gets perturbed at early stages of RD, which probably affects downstream signal processing.

### The overall proportion of GABA-positive neurons does not undergo changes in V1 of *rd1* mice

**Figure 4:**
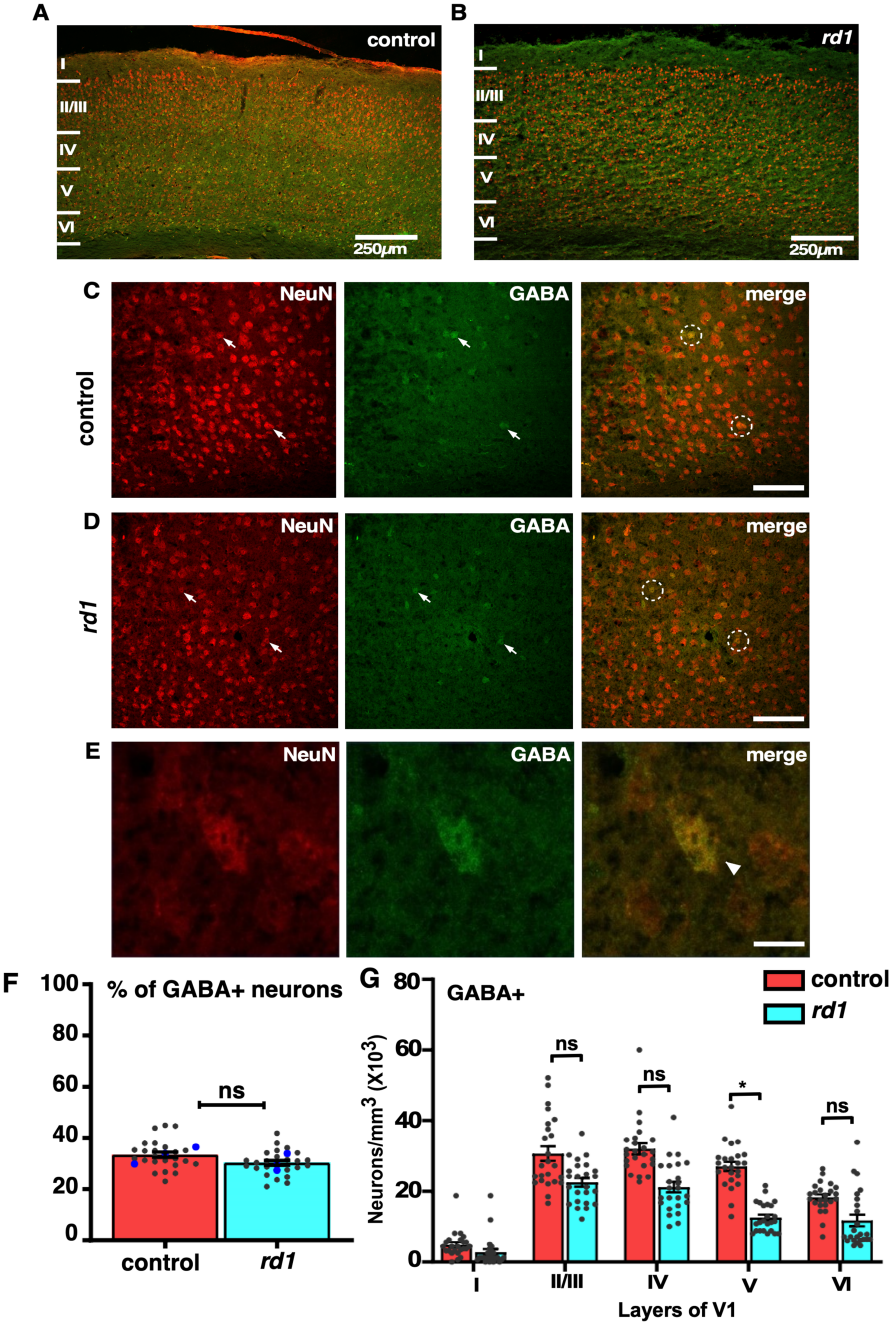
Fluorescence double labeling of NeuN (red) and GABA (green) in different cortical layers in the V1 of both control and *rd1* mice. Overview of NeuN and GABA co-labeling shows homogenous distribution across all layers of V1 in control **(A)** and *rd1* mice **(B)**. Magnified images highlight the individual staining patterns of GABA and NeuN colocalization in control **(C)** and *rd1* mice **(D)**. No change in expression was observed of GABA neurons around NeuN positive neurons. Arrows indicate neurons expressing both GABA+ and NeuN+ and white circles indicate GABA+/NeuN+ neurons. GABA colocalized with NeuN is mostly observed in cell body **(E)**. The percentage of GABA+ neurons of the total number of NeuN labeled neurons obtained from 24 coronal slices in both the strains of mice, showed no significant differences (*p* > 0.05) **(F)**. Averaged data obtained from all sections is shown in red bar for control mice and blue bar for *rd1* mice. The blue dot represents the average data obtained for individual animal and the gray dots represent data points for a single sections, providing insights into the distribution of GABAergic neurons across different animals within each strain. Graph depicting the total neuronal density in each layer of V1 for the GABA+ neurons **(G)** demonstrated no significant difference between the two genotypes for all layers except layer V. Results show mean ± SEM percentage of *n =* 3 mice of each genotype. Statistical significance was established between the two genotypes using a two-tailed Student’s *t*-test. Scalebar is shown in the bottom right panel: 250 μm **(A,B)** 25 μm **(C,D)**, 10 μm **(E)**. Magnification: 10X **(A,B)** 40X **(C–E)**.

Although absence of retinal input does not affect neuronal distribution in V1 of *rd1* mice, however, previously it has been reported that degeneration causes an increase in the inhibitory drive to improve signal quality of degraded input (Bhattacharyya, 2022). Information processing in V1 is mediated by excitatory and inhibitory circuits where GABA interneurons play an important role in balancing cortical activity (Swanson and Maffei, 2019). To determine the distribution of GABA+ neurons at an early stage of RD in cortical sections of V1 we performed immunofluorescence by employing double labeling using anti-GABA and anti-NeuN antibodies and imaged those sections using confocal microscopy (Figures 4A,B). We found widespread expression across V1 and a large proportion of NeuN+ neurons colocalizing with GABA+ neurons in both control and *rd1* mice (Figures 4C,D). The expression of NeuN was evident prominently in the cell bodies of all cortical neurons (Figure 4E). Next, we scored for GABA+ neurons among total population of NeuN+ neurons. Quantification revealed ~34% of GABA+ neurons in control sections, whereas rd1 displayed ~29% of GABA+ neurons that were statistically insignificant [control vs. *rd1*, 34 ± 3% vs. 29 ± 2%, (*p* > 0.05)] (Figure 4F). The proportion of GABA+ cells in V1 of *rd1* and control mice showed slight differences in layer IV and layer V but not in other layers: layer I control vs. *rd1*, 4,713 ± 1,453 vs. 4,069 ± 1,408, layer II/III control vs. *rd1*, 30,936 ± 3,800 vs. 27,543 ± 2,591, layer IV control vs. *rd1*, 32,367 ± 3,710 vs. 27,303 ± 2,983, layer V control vs. *rd1*, 26,816 ± 3,299 vs. 17,120 ± 1,636, layer VI control vs. *rd1*, 18,133 ± 2,133 vs. 11,595 ± 1,646 (*p* > 0.05) (Figure 4G). These results indicate that GABA expressing neurons are modestly affected by RD.

### The colocalization of GABA and PV is not affected by retinal degeneration throughout V1

**Figure 5:**
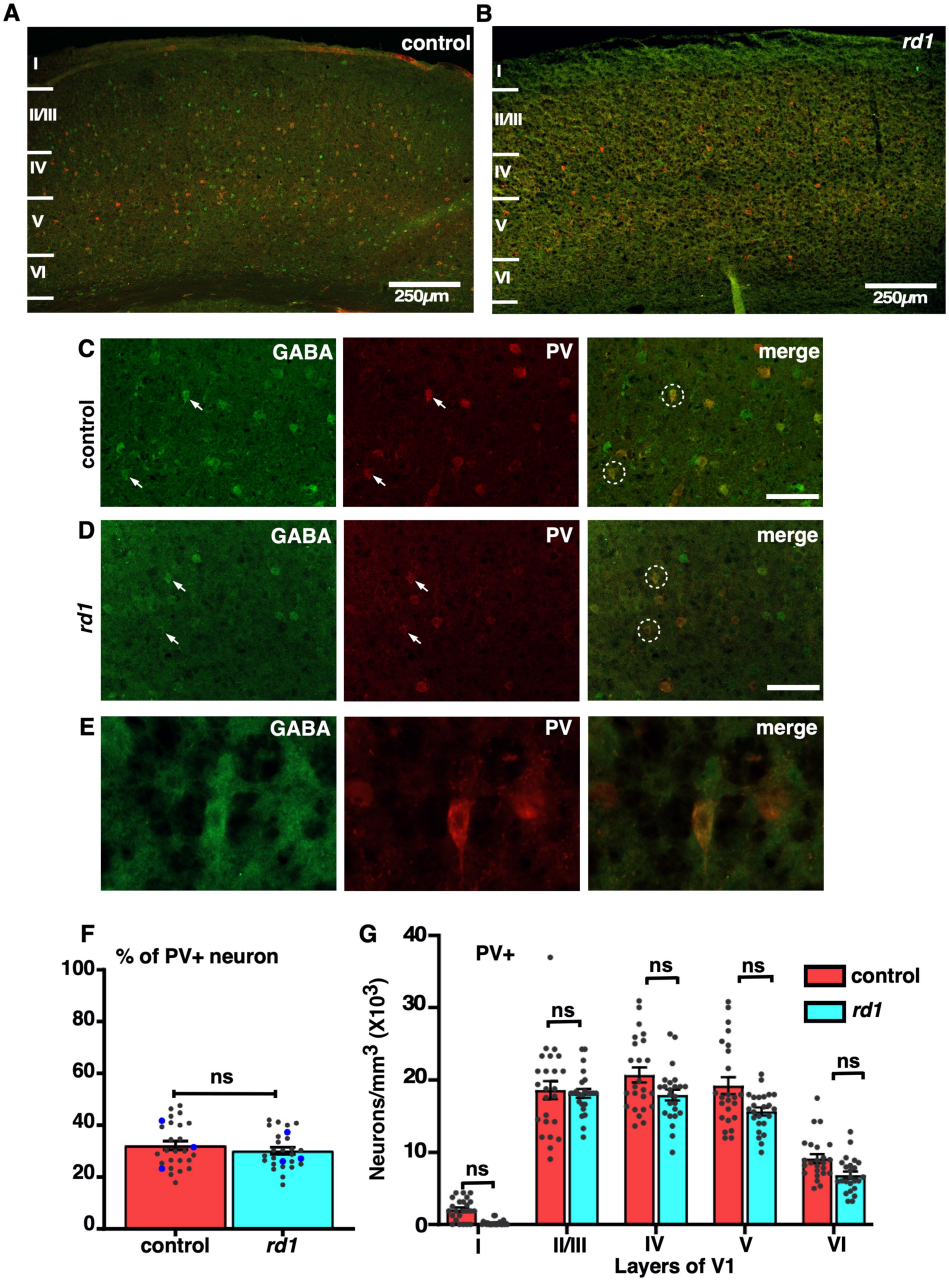
Representative confocal images of GABA (green) and PV (red) in control and *rd1* mice showing the overall distribution in V1 **(A,B)**. Magnified images of coronal sections through V1 showing PV expressed in GABAergic neurons in control **(C)** and *rd1* mice **(D)**. White arrows point to neurons expressing either GABA+ neurons or PV+ neurons in control and *rd1*. White circles show PV+/GABA+ neurons. High-power photomicrograph of an example neuron depicting the colocalization in the cell body **(E)**. The percentage of PV+ neurons in V1 of control and *rd1* were similar and showed no significant differences, (*p* > 0.05) **(F)**. Results show mean ± SEM of *n =* 3 mice of each genotype. The blue dot represents the average from individual animals and gray dots represent values for single sections. Graph depicting the total neuronal density in each layer of V1 for the PV+ neurons **(G)** demonstrated no significant difference between the two genotypes. Statistical significance was established between the two genotypes using a two-tailed Student’s *t*-test. Scalebar is shown in the bottom right panel: 250 μm **(A,B)**, 25 μm **(C,D)**, 10 μm **(E)**. Magnification: 10X **(A,B)** 40X **(C–E)**.

We next assessed the effect of RD on two major subtypes of GABAergic neurons, PV and SST. Both PV and SST are important subclasses of inhibitory GABAergic neurons having different anatomical and physiological properties (Rudy et al., 2011). Previous electrophysiological studies have demonstrated an increase in spontaneous activity and reduced capacity to discriminate visual stimulus (Chen et al., 2020; Chen et al., 2016). PV neurons represent a major class of non-overlapping interneurons responsible for modulating the activity of pyramidal neurons projecting to other layers of the cortex through perisomatic inhibition (Song et al., 2021). To examine the effect of RD on PV neurons, we performed double labeling of GABA and PV in the V1 cortical sections. We found that the distribution of PV+ neurons was slightly different in all layers. Their localization was pronounced mostly in layer IV, V, and II/III, in control and a similar trend was observed in the *rd1* mice consistent with previous findings (Goldshmit et al., 2010; Gonchar et al., 2007) (Figures 5A,B). The density of PV+ neurons was relatively less in deeper layers. Higher magnification images revealed a majority of GABA expressing neurons colocalizing with PV in both control and *rd1* mice (Figures 5C,D). The expression of PV was mostly observed in the perikarya and sometimes in the initial segment of the axons (Figure 5E). We observed few labeled cells in layer I of control animals which was completely absent in *rd1* animal. Quantitative analysis did not reveal any notable differences in the overall density of PV immunopositive neurons between the two strains (control vs. *rd1*, 31 ± 4% vs. 27 ± 1%, *p* > 0.05) (Figure 5F). Moreover, the proportion of PV+ cells in V1 of *rd1* and control mice did not show any significant layer specific differences: layer II/III control vs. *rd1*,18,600 ± 1,555 vs. 16,919 ± 2,645, layer IV control vs. *rd1*, 20,784 ± 1,376 vs. 17,707 ± 2,146, layer V control vs. *rd1*, 19,811 ± 1,688 vs. 16,026 ± 1,409, layer VI control vs. *rd1*, 9,209 ± 950 vs. 6,865 ± 1,219 (*p* > 0.05) (Figure 5G). These results suggest that early stages of RD perhaps does not affect the density of distribution of PV+ neurons in the layers of V1.

### Laminar differences in the colocalization of GABA and SST in *rd1* animals

**Figure 6:**
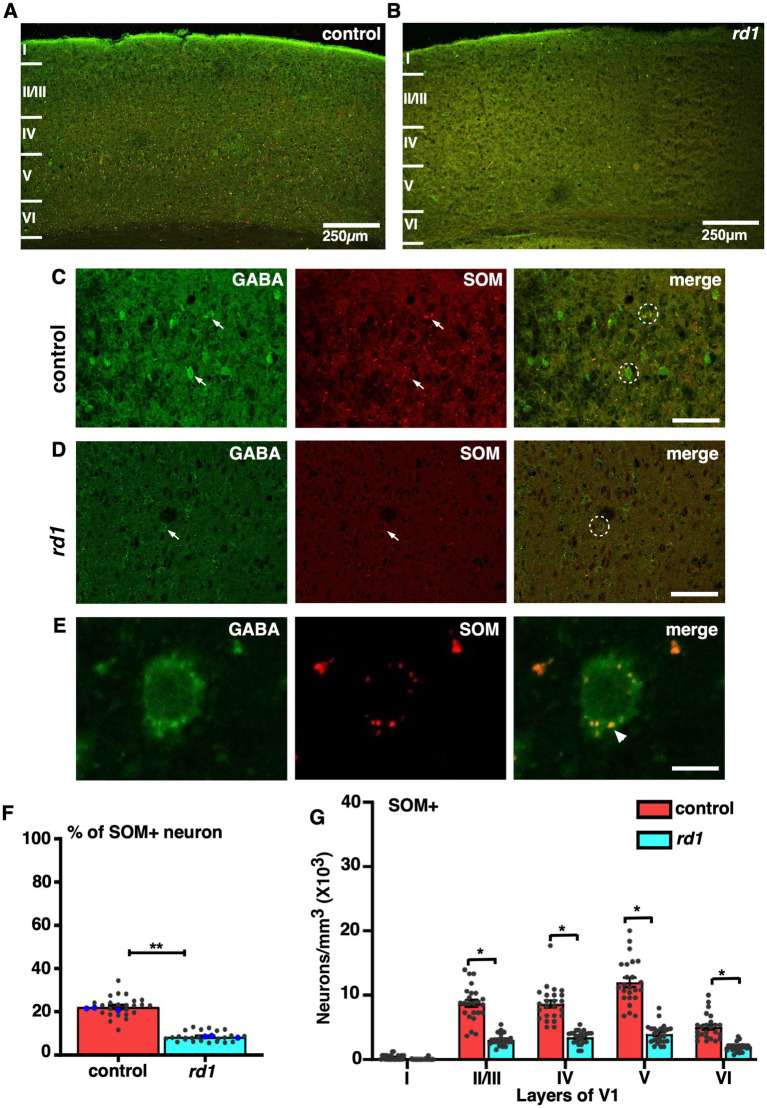
Representative confocal images of GABA (green) and SST (red) in control and *rd1* mice showing the overall distribution in V1 **(A,B)**. Magnified images of coronal sections through V1 showing SST expressed in GABAergic neurons in control **(C)** and *rd1* mice **(D)**. White arrows point to neurons expressing either GABA+ neurons or SST+ neurons in control and *rd1*. White circles show PV+/GABA+ neurons. High-power photomicrograph of an example neuron depicting the colocalization in the cell body **(E)**. The percentage of SOM+ neurons in V1 of control and *rd1* showed an overall significant decrease (*p* < 0.01) **(F)**. Results show mean ± SEM of *n =* 3 mice of each genotype. The blue dot represents the average data obtained for each animal and the gray dots represent data points for single sections. Graph depicting the total neuronal density **(G)** in each layer of V1 for the SOM+ neurons demonstrated a significant difference between the two genotypes (*p* < 0.05). Statistical significance was established between the two genotypes using a two-tailed Student’s *t*-test. Scalebar is shown in bottom right panel: 250 μm **(A,B)**, 25 μm **(C,D)**, 10 μm **(E)**. Magnification: 10X **(A,B)** 40X **(C–E)**.

We next examined the localization of SST, a GABAergic neuron that differ in their morphology and physiology depending on its layer specific expression and modulate cortical processing by dendritic inhibition (Song et al., 2021). Co-immunofluorescence staining of GABA and SST revealed a prominent expression in layer V followed by layer IV and II/III in control (Figure 6A) in accordance with previous studies (Gonchar et al., 2007). Remarkably, immunostaining for SST+ neurons in *rd1* animals showed a significant reduction in the number of cells that colocalized with GABA in all layers compared to control (Figures 6B–D). The expression of SST was observed in the distal region of the cell body (Figure 6E). The total proportion of SST+ cells in V1 of *rd1* mice and control showed significant difference (control vs. *rd1*, 22 ± 1% vs. 8 ± 1%, *p* = 0.004) (Figure 6F). Quantitative analysis revealed a significant decrease in colocalization between SST and GABA in *rd1* animals: layers II/III showed a mean decrease of 63%, while layers IV and V1 exhibited a decrease of 61%. Density of SST+ neurons in layer II/III: control vs. *rd1*, 8,674 ± 1,053, vs. 3,148 ± 411, layer IV: control vs. *rd1*, 8,958 ± 998, vs. 3,459 ± 406, layer VI: control vs. *rd1*, 5,044 ± 681, vs. 1924 ± 304. Layer V had a relatively higher decrease of 67% in comparison to control (control vs. *rd1*, 12,083 ± 1,004 vs. 3,977 ± 620) *p* < 0.05 (Figure 6G).

**Figure 7:**
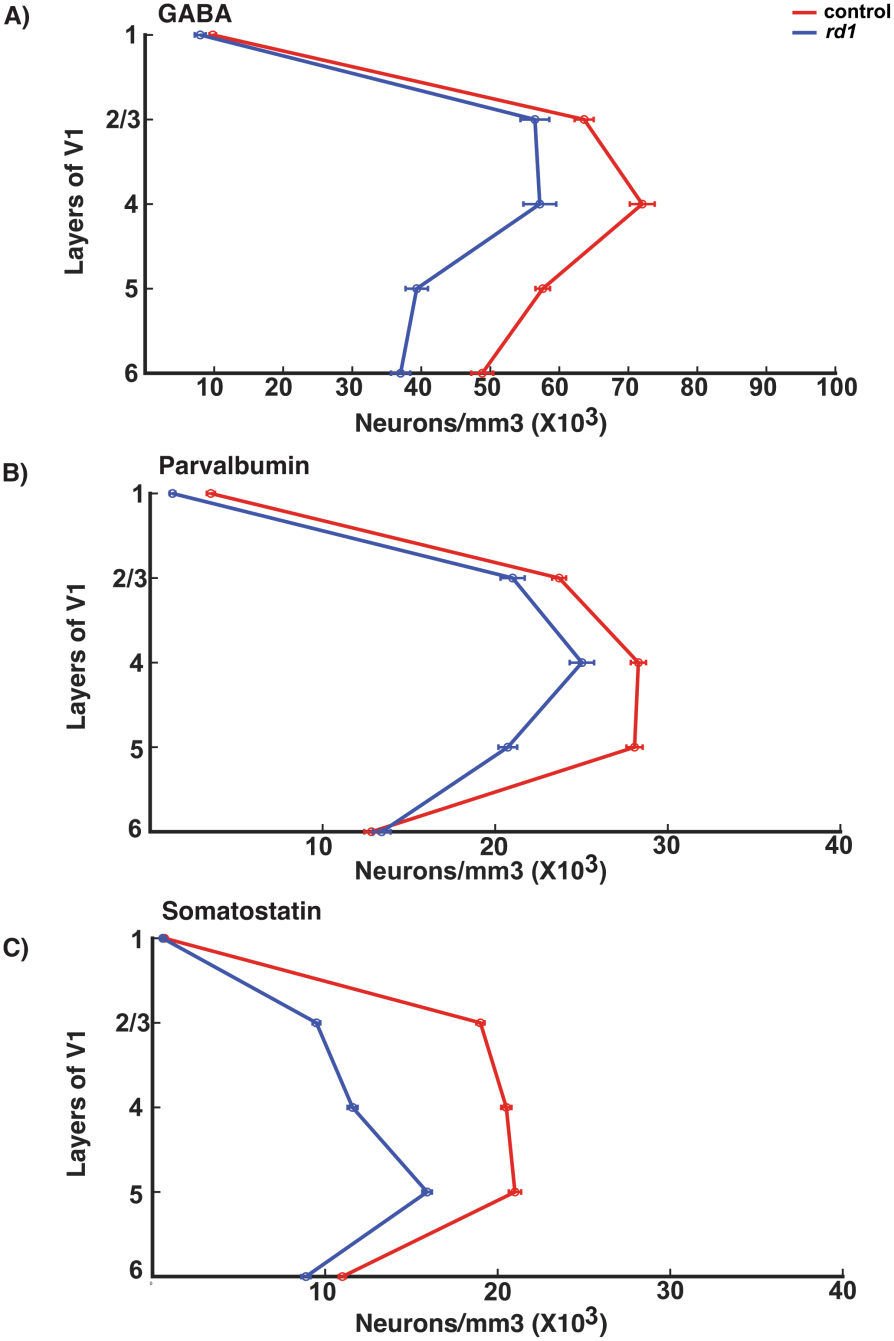
Laminar distribution of GABA, PV and SOM neurons in the V1 of control (red line) and *rd1* (blue line) mice **(A–C)**. The x-axis represents the total neuronal density, while the y-axis delineates the layers within the V1. GABAergic neurons dominate in layer IV > II/III > V > VI and less in layer I. In *rd1* mice there is a similar trend of the distribution of neurons with moderate decrease. PV neurons represent the major class of interneurons dominating mostly in layer IV > V > II/III and barely present in layer I. In *rd1* mice, the distribution and the number of PV neurons does not exhibit any changes. The population of SST neurons has prominent expression in layer V > IV > II/III which decreases during photoreceptor degeneration.

We also found that the general distribution of neuronal density of GABA neurons across all layers showed highest density in middle and upper layers (layer IV > II/III > V) and less in layer VI and I consistent with previous literature (Gonchar and Burkhalter, 1997). This distribution was similar and only modestly reduced in *rd1* animals (Figure 7A). PV neurons across all layers of V1 showed a similar pattern in both control and degenerated mice implicating PV expression is unaffected by gradual death of photoreceptors (Figure 7B). However, laminar distribution of neuronal density of SST neurons showed more concentrated neurons in the upper and middle layers (II/III/IV/V) that becomes largely reduced in degenerated mice indicating that the expression of SST is susceptible to lack of retinal input (Figure 7C). Previous studies have shown that expression of SST is cyclic AMP -responsive element binding protein (CREB) dependent and regulated by visual activity, and hence reduced activity could possibly contribute to decreased expression of SST and not necessarily cell death (Urban-Ciecko and Barth, 2016; Ampofo et al., 2020).

## Discussion

**Figure 8:**
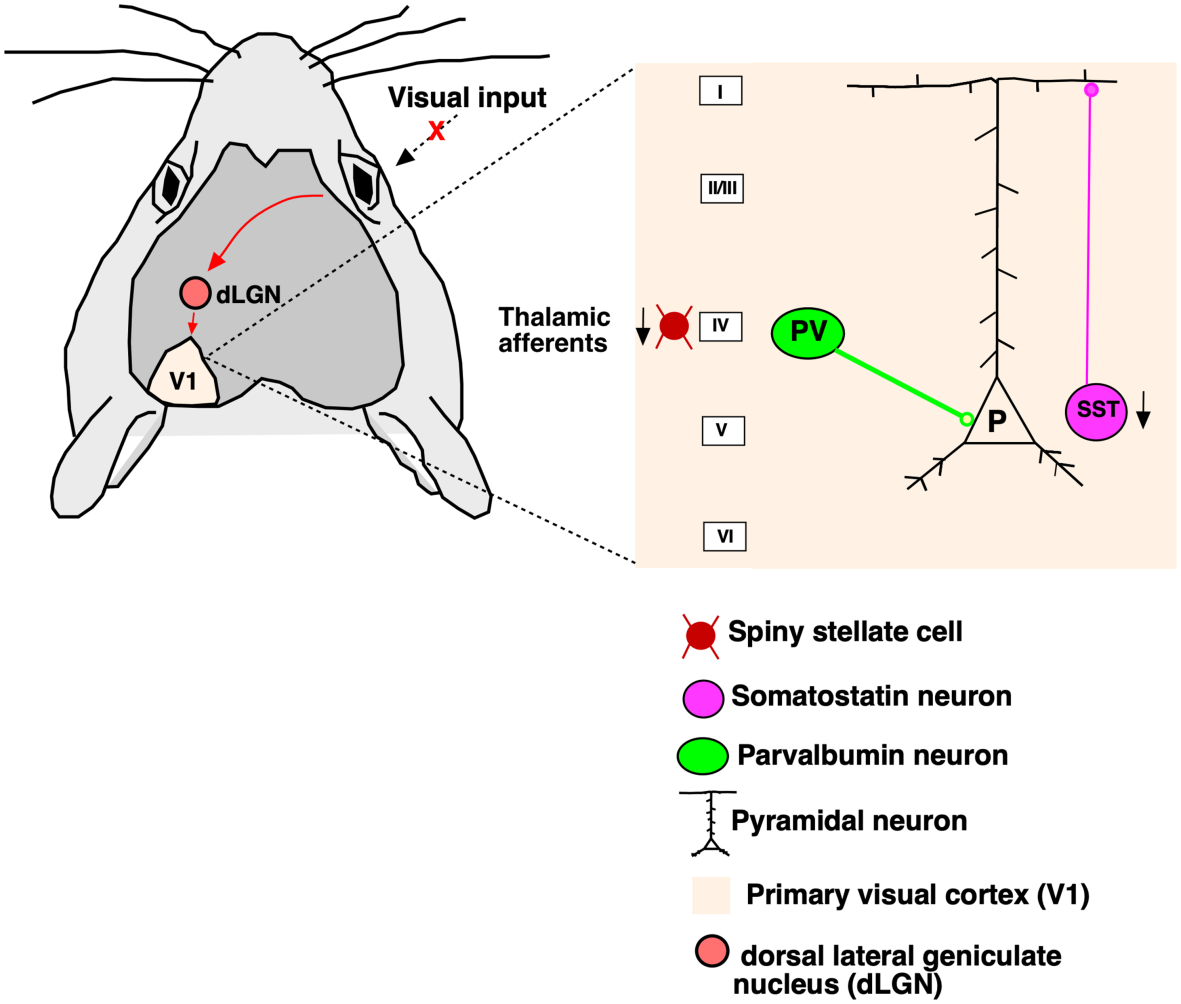
Simplified diagram of the V1 cortical circuit. The visual input from the eye travels to the dLGN and further to contralateral V1 (red arrows). Upon degeneration of photoreceptors in RP changes unfold in the V1. Thalamocortical afferents are greatly affected in the absence of visual stimuli. Parvalbumin neurons that target the somatic compartment of the pyramidal neurons are not affected by degeneration. Somatostatin neurons which target the distal dendrites are substantially decreased in all layers of cortex.

Here, we demonstrate the effects of RD on the GABAergic neurons and its two important subclasses (PV, SST) using immunohistochemical staining, confocal microscopy and cell quantification in control and *rd1* animal brain samples. Our data support the notion that PV+ neurons remain unaffected by RD, whereas SST+ neurons are significantly reduced in all layers of V1 which is illustrated with a simplified schematic representation (Figure 8). We demonstrate decreased immunolabeling of VGLUT2 in layer IV and its reduced expression by Western Blot analysis indicating downregulation of the vesicular transporter in the thalamocortical terminals that might impair glutamatergic transmission. The *rd1* model has been consistently used for decades in restoring natural vision through optogenetic vision restoration, but so far, the recovery of vision has been mostly suboptimal (Berry et al., 2017; Katada et al., 2023; Gauvain et al., 2021; Chen et al., 2022; Sahel et al., 2021; Kalloniatis et al., 2016; Gaub et al., 2015). The limitation of successful restoration could be due to changes in the structures downstream of the retina. Until now, to the best of our knowledge, there are no studies that have investigated the changes in inhibitory neurons underlying degeneration in *rd1* mice. The higher visual areas, such as V1, is susceptible to retinal remodeling and altered visual input conveyed by the impaired retina (Caravaca-Rodriguez et al., 2022; Ferreira et al., 2017). Consistent with previous data, we observed the absence of photoreceptor layer at P40 while the inner retinal layers were found to be intact (Santos et al., 1997). The rate of degeneration in *rd1* is rapid in contrast to *rd10*, another mouse model of RD where a thin layer of outer nuclear layer still persists at P32 (Pennesi et al., 2012; Chang et al., 2007). Strikingly, we observed no changes in the V1 laminar cortical organization in the *rd1* mice measured by thickness, distribution of individual layers and total number of neurons. These results are in line with previous studies in congenital blind mice where no gross changes in structure and lamination were reported (Goldshmit et al., 2010). However, in humans with RP there are significant changes in the gray matter volume and the association cortices confined to the occipital cortex of patients (Rita Machado et al., 2017). Consistent with this, a noticeable decrease in brain weight and cortical thickness at both early and late stages of degeneration have been reported in rat model (P23H) of RD (Martinez-Galan et al., 2022). However, in another study performed on a different rat model (S334) the researchers have not reported of any significant changes of depth in V1 resulting from degeneration (Chen et al., 2016). Hence, the degeneration of the photoreceptors might not necessarily alter the overall thickness of the cortex or the laminar organization in V1.

GABAergic neurons comprise about 15–25% of all cortical neurons of which specific subtypes are recruited for feature selectivity by the V1 neurons (Song et al., 2021; Jones, 1993). We used double immunolabeling to count the total GABAergic population (GABA+) of neurons as a subpopulation of all neurons (NeuN+). Our data indicate that GABA+ neurons in the V1 of control and *rd1* mice correspond to 34 and 29%, respectively. Thus, the proportion of GABA+ neurons in the V1 cortical region of *rd1* is almost similar to that of normal seeing mice and does not undergo changes due to RD. These results are consistent with recent reports where cortex retains GABAergic neurotransmission to avoid deterioration of visual function (Pietra et al., 2021). However, in aging the proportion of inhibitory neurons becomes significantly reduced causing functional degradation (Ding et al., 2017). A previous study reported an increased ratio of GABAergic neurons in the lateral geniculate nucleus (LGN) following long term lesion of V1 suggesting that the GABAergic neurons were less susceptible to retrograde degeneration (Atapour et al., 2021).

Further, we investigated the effects of RD on PV and SST subpopulations of GABAergic interneurons in the cortical microcircuit of *rd1* animals. PV and SST are non-overlapping chemically distinct groups of neurons and comprise about 40 and 30% of cortical interneurons (Druga et al., 2023) (Figure 8). These cells target the soma and distal dendrite region of the pyramidal neurons (Roux and Buzsaki, 2015). Our data also reveal similar expression of PV and SST comprising about 31 and 22% in control animals whereas 27 and 8% in *rd1* animals, respectively. There was a significant reduction in the SST+ population of neurons between control and *rd1* whereas PV+ neurons remained unaltered. The proportion and distribution of PV neurons in V1 agree with previous studies that account 39% of total population of GABA neurons in the adult mouse visual cortex (Gonchar et al., 2007). It is interesting to note that PV neurons undergo substantial changes in mice that are deprived of visual input since birth (Goldshmit et al., 2010). The reduction of PV neurons in congenital blind animals imply that their expression is dependent on visual input while *rd1* mice that are not completely devoid of visual activity retain PV neurons. It is therefore very likely that these neurons are recruited by the visual system to improve the signal quality of degraded input by increasing the inhibitory drive to compensate the excitation/inhibition imbalance (Bhattacharyya, 2022; Bhattacharyya et al., 2013). This could be a plausible reason for the increase in spontaneous activity reported in rat model of RD (Wang et al., 2016; Wang et al., 2018; Chen et al., 2020).

Our findings for the SST+ neurons are persistent with the multiple labeling study of mouse cerebral cortex comprising 24% of total GABAergic neurons (Gonchar et al., 2007). Optogenetic studies have shown that SST neurons can cause both dendritic inhibition and disinhibition of excitatory pyramidal neurons via GABAergic transmission. This enables them to alter stimulus selectivity which is a key attribute of V1 neurons (Song et al., 2021; Wilson et al., 2012). Several electrophysiological studies in degenerated rats have demonstrated diminished orientation selectivity and inability to discriminate stimuli under different contrast conditions (Chen et al., 2020; Chen et al., 2016). Therefore, during RD, a significant decrease in the population of SST+ neurons in different layers might manifest in deterioration of visual discrimination ability in V1.

The current findings provide direct evidence of decreased VGLUT2 labeling in mouse V1 and its reduced expression levels at early stage of RD. In contrast, immunolabeling in rats have reported of increased expression of VGLUT2 in advanced stages (P230) but not in early stages (P30) (Martinez-Galan et al., 2022). Our estimates of VGLUT2 at early ages of *rd1* (P30) could arise from rapid pace of degeneration unlike *rd10* which has a slower onset. Interestingly, the alterations suggest plastic changes that happen between early and intermediate stages which may flatten or decrease with advanced age.

Taken together, our results demonstrate the implications of RD on the distribution of GABAergic inhibitory neurons and its subtypes, PV and SST in addition to decreased expression of VGLUT2 in thalamocortical afferents in V1 of *rd1* mice. Our studies provide insights for the altered excitation/inhibition imbalance observed in visual cortex physiology. Somatostatin has modulatory effect on the cortex and receives direct afferents from the basal forebrain (Do et al., 2016). Hence, stimulation of the basal forebrain and/or SST treatment by exogenous application in V1 cortical circuit might prove beneficial in balancing excitatory synaptic transmission and enhancing orientation selectivity (Bhattacharyya et al., 2013; Song et al., 2020). Further, dissecting the functional role of the PV-SST circuit via optogenetic manipulation in mice model of RP will provide opportunities to develop better strategies for functional recovery.

## Materials and methods

### Animals

Experiments were carried out on the V1 of C57/BL6 (control) and C3H/HeJ (*rd1; r*etinal degeneration) mice weighing 25–30 g. The C3H/HeJ mice (Strain # 000659) were obtained from the Jackson Laboratory, USA. The animal house facility maintained a temperature range of 18–25°C and followed a 12-h light–dark cycle. Three animals each from the control and *rd1* group were used for all experiments. The research strictly followed the ethical rules established by the Institutional Animal Ethics Committee Approval (IAEC), of Amity University, Noida ensuring that animals were treated humanely and ethically throughout the study.

### Tissue processing

Animals were anesthetized with Thiopentone sodium (50 mg/kg) via intraperitoneal injection and perfused with PBS, followed by 4% PFA. Eyes were then dissected leaving the optic nerve attached to the retina and brains were carefully removed from the skull, washed in PBS and both eyes and brain were postfixed in 4% PFA overnight at 4°C. The tissue of both the eye and brain was embedded in OCT compound after being successively incubated in 10, 20, and 30% sucrose solutions followed by sectioning using a Cryostat. Eyeball sections were 10 μm sagittal, while brain sections were 20 μm coronal. The sections were taken on 1% gelatin-coated slides and stored at −20°C before immunohistochemical analysis.

### Immunohistochemistry

#### Retinal histology

Toluidine blue staining was used for comparing retinal sections from *rd1* and control mice. Frozen retinal tissue sections were rinsed with PBS until OCT was properly washed. Following this, the sections were immersed in 0.1% toluidine blue solution for 1 min, excess stain was rinsed off with distilled water (dH20). Following rinsing, they were dehydrated in 70 and 95% ethanol for 30 s. Slides were further cleared after dipping in 3 changes of xylene for 30 s and were kept for air drying. Slides were cover-slipped using DPX as a mounting medium.

#### Immunofluorescence

All sections were subjected to standard immunohistochemical protocols, which included appropriate negative and positive controls. Sections at the anterior posteriority (AP) of −2.5 mm to −3.0 mm containing an area of V1 were considered for the experiments. Slides were brought to room temperature and washed with PBS (pH 7.4) to remove OCT (3*10 min). The sections were immersed in a solution of 3% H2O2 and methanol for 20 min to block endogenous peroxidase activity. Following this, sections were washed with distilled water and then blocked for non-specific binding by incubating in 5% animal serum in PBS-T for 30–40 min at room temperature. The sections were then incubated in respective primary antibody dilution and were left overnight at 4°C. Primary antibodies that were used to detect specific neuronal cell populations are: mouse anti-neuronal Nuclei (NeuN) (1:1000, Abcam, ab104224), Rabbit anti-gamma Amino Butyric Acid (GABA) (1:1000, Sigma, A2052), Mouse Monoclonal Anti-Parvalbumin (Anti-PV) (1:1000, Sigma, P3088), Rat anti-somatostatin (SOM) (1:1000, Invitrogen™, MA5-16987). After incubation sections were washed twice with PBS and were then incubated in fluorescent-tagged secondary antibodies for 2 h at room temperature. The secondary antibodies that were used are: Alexa Fluor 564 (1:700, Invitrogen, A-11004), Goat anti-Rabbit IgG (H + L) Cross-Adsorbed Secondary Antibody, Alexa Fluor 488 (1:1500, Invitrogen, A-11008), Goat anti-Rat IgG (H + L) Cross-Adsorbed Secondary Antibody, Alexa Fluor 543 (1:700, Invitrogen, A11081) (Table 1). After several washes with PBS (3*10 min) sections were incubated in DAPI (4′, 6-diamidino-2-phenylindole) staining solution (1,1,000) prepared in PBS and were then washed with PBS again (3*10 min). Stained tissue sections were air-dried, dehydrated in ethanol, cleared in xylene, and were cover-slipped with a DPX mounting medium for microscopic examination (Table 1).

**Table 1:** List of antibodies used for immunofluorescence.

Antibody name	Supplier details	Catalog number	Dilution used
Primary antibodies			
Anti-VGLUT2 (Vesicular glutamate 2 transporter)	Invitrogen™ Rabbit polyclonal antibody	PA5119621	1:2500
Anti- GABA	Sigma-Aldrich Rabbit polyclonal antibody	A2505	1: 1000
Anti-Parvalbumin	Sigma-Aldrich Mouse monoclonal antibody	P3088	1: 1000
Anti-Somatostatin	Invitrogen™ Rat monoclonal antibody	MA5-16987	1: 1000
NeUN (E4M5P)	Cell signaling technology Mouse monoclonal antibody	mAb 94403	1: 1000
beta Actin	Invitrogen™ Mouse monoclonal antibody	AM4302	1:3000
Secondary antibodies			
Goat anti-Mouse Ig (H+L) cross-Adsorbed, Alexa Fluor™ 568	Invitrogen™ Polyclonal antibody	A-11004	1:800
Goat anti-Rat IgG (H+L) Cross-Adsorbed, Alexa Fluor™ 546	Invitrogen™ Polyclonal antibody	A-11081	1:800
Goat anti-Rabbit IgG (H+L) Cross-Adsorbed, Alexa Fluor™ 488	Invitrogen™ polyclonal antibody	A-11008	1:1500
Goat anti-Rabbit IgG (H+L) Secondary Antibody, HRP	Invitrogen™ Polyclonal antibody	31460	1:5000
Goat Anti-Mouse IgG (H + L)-HRP Conjugate	BIO-RAD Polyclonal antibody	**1706516**	1:5000

In addition, we performed VGluT2 staining to identify the thalamocortical afferents. In this protocol, we prepared a primary antibody solution of VGluT2 (Anti VGluT2) (1:200, Invitrogen, PA5119621) and kept in incubation for 24 h at 4°C. After washing with PBS (3*10 min) sections were kept for secondary antibody incubation [Alexa Fluor 488 (1:1500, Invitrogen, A-11008)] in room temperature for 2 h (Table 1). After several washes, sections were incubated in DAPI solution (1:1000) and cover slipped.

#### Microscopy and data analysis

Images were captured with excitation filters suited to the 488, 564, and 543 nm laser lines, and image resolution per frame was 1,024 × 1,024 pixels or 2046 × 2046 pixels using a confocal laser scanning microscope (Leica TCS SP8) with objectives 10x, 20x, and 40x. For a thorough analysis, we took pictures of 8 sections per animal (*n =* 3). Any sections having pale staining areas or gaps were not used for analysis. Quantitative analysis for cell counting was done on pictures taken at 20x magnification.

V1 was identified using the coordinates from the Paxinos brain atlas (Franklin and Paxinos, 2013), and was divided into 6 cortical layers (I, II-III, IV, V, VI) similar to previous studies (Gonchar et al., 2007). Immunolabeled V1 neurons were sampled in rectangular counting frames (804.19 μm) with varied heights (100–200 μm) based on the area of each layer (I-VI) (Atapour et al., 2019). Neuronal quantification was performed using ImageJ software having the cell counter plugin on sections of *rd1* and control mice immunostained for NeuN, GABA, PV, and SOM (Rueden et al., 2017). Counting included all neurons with clear fluorescence, regardless of intensity, and the same procedure was followed for counting GABA, PV, and SOM neurons. Thus, we were able to determine the quantity of single- (e.g., PV alone, GABA only, NeuN only, SOM only) and double-labeled (e.g., PV + GABA, NeuN + GABA, GABA + SOM) neurons. We determined the percentage of colocalization based on the ratio of PV+ cells that express GABA and the proportion of SOM+ cells that colocalize with GABA.

### Estimating neuronal density

We calculated the number of neurons with the help of the ImageJ cell counter plugin. The density of the neuronal population is done using the following formula:


$$\mathrm{Density}=\frac{\mathrm{Cell}\;\mathrm{count}\;}{\mathrm{Area}\;\mathrm{of}\;\mathrm{sampling}\;\mathrm{frame}*\mathrm{Thickness}\;\mathrm{of}\;\mathrm{section}\;}$$

After calculating the neuronal density for each layer based on their sampling frame size, we combined the results from each section of different animals for further statistical analysis. To quantify VGLUT2, images from both mice strains were taken using a confocal microscope, with the same surface area, and the same number of images were considered for both strains. Fiji (ImageJ) (Schindelin et al., 2012), was used to process the images, converting them to an 8-bit grayscale to standardize the pixel values and make it easier to remove the background noise. The pixel intensity range was kept consistent at 0–255 for all images. We then measured the average pixel intensity (Mean Gray value, MGV) for each image to quantify VGLUT2 labeling.

### Western Blot

Animals were sacrificed by an intraperitoneal injection of thiopentone sodium (50 mg/kg). The brain was immediately removed, and the visual cortex was dissected and stored in PBS at −20°C until use. We compared control and *rd1* mice (*n =* 3). Tissues were homogenized in ice-cold Tris-EDTA lysis buffer. Homogenates were centrifuged at 13,000 rpm for 15 min at 4°C. Supernatants were stored at −20°C. Protein concentration was determined by BCA Assay (#A55864). Un-boiled protein extracts (15 μg) were electrophoresed on 10% SDS polyacrylamide gels using the mini-PROTEAN III system (Bio-Rad) for over 90 min at a constant 25-30A. Gels were transferred to PVDF membrane for 70 min at 110 V. Immunodetection was performed by first blocking the membranes with 5% skimmed milk in TBST (TBST: 50 mM Tris, 200 mM NaCl, 0.1%Tween 20) for 1 h at room temperature (RT). The membranes were incubated in a primary antibody, VGLUT2 (1:2500, Invitrogen, #PA5119621) in 5% skimmed milk in TBST overnight at 4°C followed by washing with TBST and incubation in HRP-conjugated secondary anti-rabbit antibody (1:5000, Sigma-Aldrich, #A9169) for 1 h at RT. The membranes were washed with TBST and developed using chemiluminescence assay (Pierce ECL Western Blotting Substrate) and scanned in FluorChem Western Blot Imaging System. The membranes were washed, blocked and incubated in anti-*β*-actin antibody (1:2500, Invitrogen, #AM4302) for 1 h at RT. This was followed by incubation in HRP-tagged anti-mouse antibody (1:5000, Invitrogen, #31460) for 1 h at RT after which they were developed and scanned. Densitometry analysis was performed using ImageJ software.

### Statistical analysis

Student’s *t*-tests were used for all the comparisons between *rd1* and control animals. Results presented here are displayed as the mean ± standard error of the mean (SEM). A significance level of *p* < 0.05 was adopted to assess statistical significance.

## Data Availability

The original contributions presented in the study are included in the article/further inquiries can be directed to the corresponding author.
